# Social Network Analysis of Intensive Care Unit Health Care Professionals Measured by Wearable Sociometric Badges: Longitudinal Observational Study

**DOI:** 10.2196/23184

**Published:** 2020-12-31

**Authors:** Eiji Kawamoto, Asami Ito-Masui, Ryo Esumi, Mami Ito, Noriko Mizutani, Tomoyo Hayashi, Hiroshi Imai, Motomu Shimaoka

**Affiliations:** 1 Department of Emergency and Disaster Medicine Graduate School of Medicine Mie University Tsu-City Japan; 2 Department of Molecular Pathobiology and Cell Adhesion Biology Graduate School of Medicine Mie University Tsu-City Japan; 3 Emergency and Critical Care Center Mie University Hospital Tsu-City Japan

**Keywords:** wearable, interprofessional communication, clinician interaction, social network analysis

## Abstract

**Background:**

Use of wearable sensor technology for studying human teamwork behavior is expected to generate a better understanding of the interprofessional interactions between health care professionals.

**Objective:**

We used wearable sociometric sensor badges to study how intensive care unit (ICU) health care professionals interact and are socially connected.

**Methods:**

We studied the face-to-face interaction data of 76 healthcare professionals in the ICU at Mie University Hospital collected over 4 weeks via wearable sensors.

**Results:**

We detail the spatiotemporal distributions of staff members’ inter- and intraprofessional active face-to-face interactions, thereby generating a comprehensive visualization of who met whom, when, where, and for how long in the ICU. Social network analysis of these active interactions, concomitant with centrality measurements, revealed that nurses constitute the core members of the network, while doctors remain in the periphery.

**Conclusions:**

Our social network analysis using the comprehensive ICU interaction data obtained by wearable sensors has revealed the leading roles played by nurses within the professional communication network.

## Introduction

Knowing how people interact in the workplace is an important basis for understanding how effective collaborative behavior develops. Social network analysis can be a potentially powerful tool for systematically assessing the nature of human interactions in the workplace, thereby elucidating the overall structure of organizational behavior. This can be depicted as a sociogram, which visualizes how interactions take place, how people are connected, how relationships are formed, and how information is transferred [1]. Social network analysis has been used to study human interactions in health care settings; however, its utility in promoting communication and collaboration between health care professionals remains to be demonstrated [2]. One of the factors potentially undermining social network analysis in health care and other relevant settings is the lack of an effective technology for objectively and comprehensively acquiring data [2]. Currently, datasets for social network analysis are usually acquired manually via questionnaires, observations, and manual retrieval of electronic medical and administrative records [3,4]. These manual data acquisition methods make it difficult to carry out objective and comprehensive/continuous measurements of social network connections among medical staff. The development of novel enabling technologies designed to address this problem has long been desired. Such technologies could improve the quality of social network analysis research by identifying the targets of interventions vis-à-vis collaborative behaviors.

Wearable sociometric sensor badges have emerged as a robust enabling technology to monitor such interactions objectively and comprehensively [5]. This study used wearable sensors, which have previously been used in both health care [6] and corporate [7] settings. They are embedded with 6 infrared data association transceivers on the front of the badge that detect interpersonal interactions. These wearable sensors also contain a built-in acceleration detector, thereby enabling the measurements of bodily motions associated with verbal communication. In addition, location information is obtained by using infrared beacons set at specific locations throughout the workplace. In this way, the wearable sensors are able to collect the data on who meets whom, when, where, and for how long, gathering objective and comprehensive datasets for social network analysis.

Effective interprofessional interactions are critical to the complex clinical environments involving multidisciplinary collaborations typical of intensive care units (ICUs) [8-10]. We recently performed a feasibility study [6] using wearable sociometric sensor badges in a health care setting to collect human behavioral interaction datasets. The design of our feasibility study involved the monitoring of face-to-face interactions among 76 health care professionals in the ICU at a university hospital in a comprehensive and objective manner. We demonstrated that the wearable sensor badges were able to objectively capture the temporal dynamics of the interactions between health care professionals at work. The intensities of the interactions peaked 3 times per day, exhibiting 8-hour interval periodic changes, which mirrored the reality of the ICU (ie, 3 shift changeovers spanning the day, evening, and night shifts). However, how the health care staff interacted interprofessionally in the ICU remains to be elucidated. Thus, building on our previous feasibility study, we carried out social network analysis to investigate the nature of the interprofessional interactions conducted in the ICU.

## Methods

### Participants

This study was approved by the Mie University Human Research Ethics Committee (approval no. 2978) as previously described in our feasibility study [6]. Briefly, a clinical investigation involving 76 ICU health care professionals was carried out in the ICU at Mie University Hospital. The ICU functions as a referral center for critical patients with such conditions as high-degree burns, post–cardiac arrest, septic shock, and other life-threatening traumas. Staff members working within the ICU (physicians, nurses, nursing assistants, pharmacists, medical technicians, desk clerks, and secretaries) were included in the study. Written consent was obtained from all participants. A few staff members who preferred not to participate wore mock badges that tuned off all sensor functions in order to maintain anonymity. A total of 76 medical professionals participated in the research project: 15 attending physicians, 39 nurses, 4 senior residents, 1 resident, 4 nursing assistants, 8 medical technicians, 2 receptionists, 1 pharmacist, and 2 secretaries. All participants wore the badges on the front of their clothing for 4 weeks during their working hours, including breaks.

### Wearable Sensors and Data Acquisition

Wearable sociometric sensor badges (Business Microscope, Hitachi, Ltd, Tokyo) were used as previously described in our feasibility study of the ICU [6] and in another study of the corporate call center [7]. Briefly, the badges attached to the participants’ front pockets enable automatic and comprehensive collection of the datasets required for the social network analysis of ICU health care professionals. These badges captured the wearer’s physical movements via a 3-axis micro electro-mechanical acceleration sensor, which is used to detect individual activities. The badges also detect interpersonal interactions via 6 infrared data association transceivers on the front of the badge, as previously described [7]. Data on who met whom, when, and for how long can be collected. Location information is obtained by using infrared beacons set at specific locations within the ICU. Four infrared data association transceivers on the front of the beacon, facing at slightly different angles, create a detection range encompassing 60° horizontally and 30° vertically. The use of infrared beacons stationed in the ICU accords a 2-dimensional view of the location of the participants. With the use of these badges and the infrared beacons, the researchers were able to measure the duration and location of face-to-face communication among the study’s participants. Two individuals were considered to have “actively communicated” with each other if there was a face-to-face event between them that exceeded a predefined threshold (ie, 2 Hz) of more than 1 minute. Active face-to-face interactions were previously classified as comprising gesture-aided conversation [6,7,11,12]. To locate the position of these human interactions within the ICU, a total of 249 infrared beacons were widely placed throughout the ICU as previously described [6] in 42 functional areas in which work-related interactions occurred, including 14 beds, a central nurses’ station, conference room, consultation room, computed tomography scan control room, 4 examination-and-procedure rooms, family room, 3 laboratories, nurses’ lounge, physicians’ lounge, 2 physician stations, reception, satellite pharmacy, shower room, 4 utility rooms, and 5 storage rooms, but excluding corridors. As the infrared beacon beams, which are subject to interference by any concrete objects, travel up to 2 meters, multiple beacons were installed in each area to detect any interactions. Beacons between the nurses’ station, bedsides, and other areas were far enough apart (ie, >4 m) to prevent any overlapping measurements.

### Social Network Analysis

Using the datasets acquired by the wearable sensors, social network analysis was performed as previously described [5,7]. Social network connections are defined as face-to-face interactions between specific subjects (nodes) and are depicted as nodes connected with lines. The distances between nodes are inversely proportional to the total lengths of the face-to-face interactions between specific subjects. Centrality measurements were carried out as previously described [13,14].

## Results

### Spatiotemporal Distribution of the Active Face-to-Face Interactions

We analyzed a large data set previously collected in our earlier feasibility study that involved 76 ICU staff, each of whom worked for 160 hours on average during the 4-week period of data collection, totaling 729,600 minutes of active, person-to-person interaction. To determine exactly where the staff actively interacted with each other in the ICU ward each day, we mapped the daily-accumulated active interactions to the floor layout of the ICU during each day (from Days 1 to 30) (See Multimedia Appendix 1). The majority of the active interactions occurred in the patient area, in which several active interaction “hot spots” were identified at patient bedsides and the central nursing station. Outside of the patient area, hot spots were also observed in the nurses' lounge, the conference room, and the staff office (See Multimedia Appendix 2 and Multimedia Appendix 3).

### Profession-Specific Active Communication

**Figure 1:**
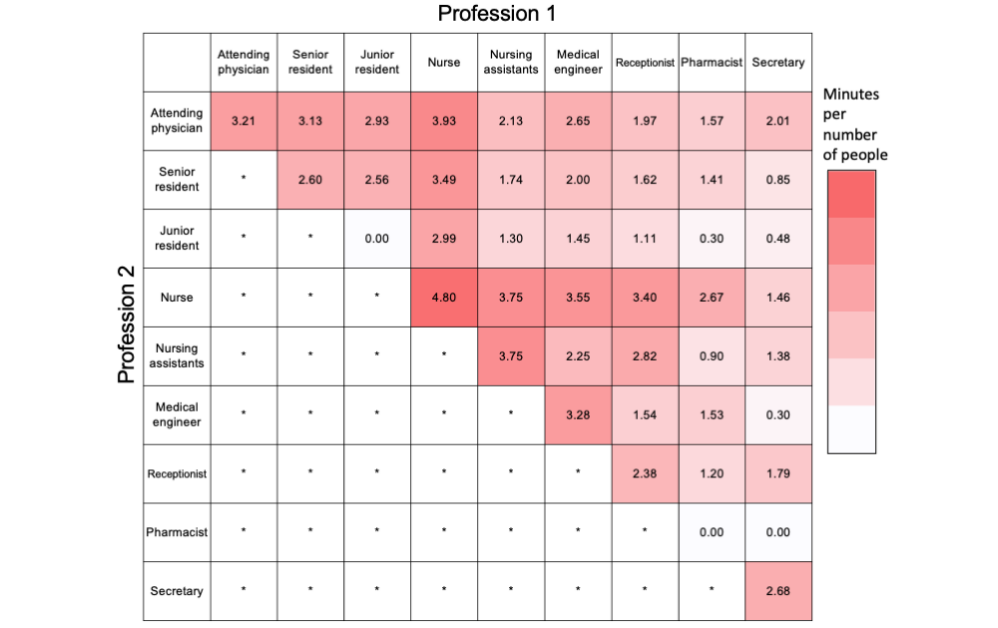
Pair preferences for active face-to-face interactions among the ICU staff. A heat map representation showing the cumulative lengths of the active face-to-face interactions (min x person) between specific pairs of professions. The data are displayed via a logarithmic scale that reflects the length of the interactions multiplied by the number of people. *Not applicable.

Next, we compared the total lengths of daily active communication per profession. The lengths of active daily interactions involving either nurses or nursing assistants were significantly greater than those of other professions (Figure 1). Nurses most often actively interacted with other nurses, and the length of their active interactions were greater than all of the other inter- and intraprofessional active interactions. Nurses also actively interacted well with many other professions (Figure 1). By contrast, nursing assistants actively interacted with each other and with nurses to a significant degree, but not with doctors. Attending physicians actively interacted mostly with nurses, followed by other attending physicians and residents. These data suggested the pivotal roles played by nurses and their interprofessional communication in the ICU.

### Communication Network Analysis

**Figure 2:**
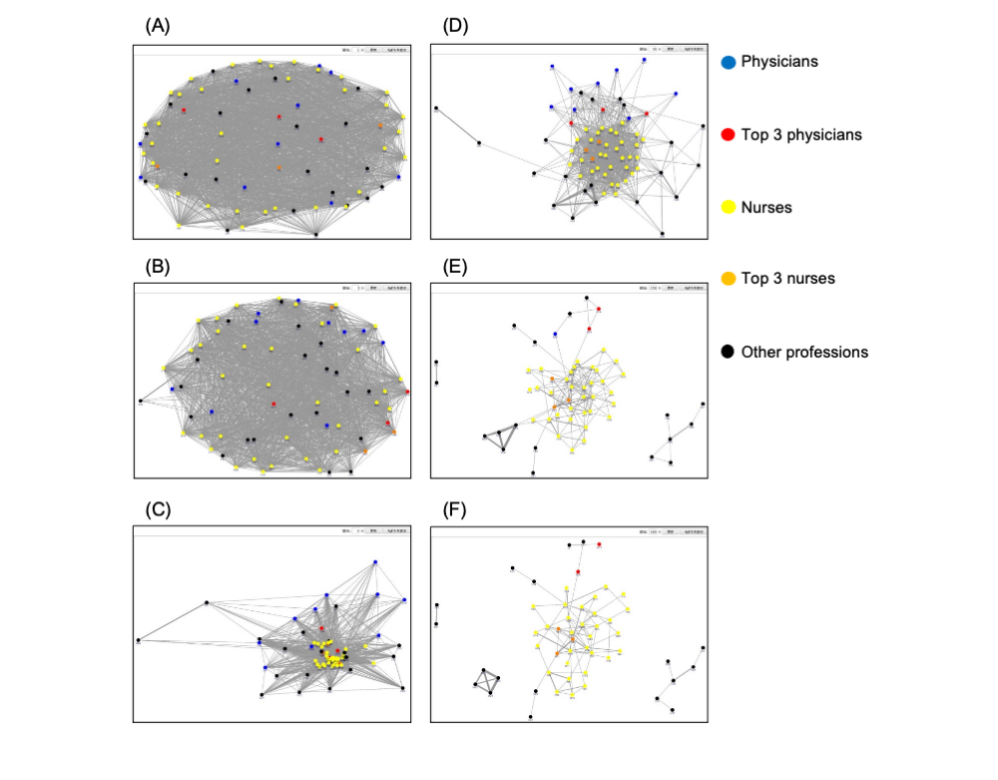
Social network analysis of active face-to-face interactions. Nodes represent individual ICU staff: blue and red, doctors; yellow and orange, nurses; black, other professions. The top 3 doctors and nurses who actively interacted the most are denoted by red and orange nodes, respectively. The following threshold values were used to define those links connecting the nodes: (A) 1, (B) 3, (C) 4, (D) 30, (E) 150, and (F) 180 minutes during the 4-week measurement period.

To better understand the roles played by nurses in interprofessional communication in the ICU, we carried out a social network analysis. We focused our analysis on nurse-physician interactions, as nurses actively interacted not only with each other, but also with physicians (Figure 2). Moreover, nurses and physicians represent the two major groups in the ICU medical team [15]. The network connectivity between ICU staff was determined by the cumulative total lengths of active face-to-face interactions. By gradually changing the threshold from 1 to 180 min in order to determine the degree of connectivity (Figures 2A to 2F), different layers of the communication network in the ICU emerged. The communication network sociograms created using a threshold of 30 min (Figure 2D) demonstrated the core/periphery network structure [16], in which nurses were centrally positioned at the core, whereas physicians and other professions were at the periphery. The sociograms of the core group members contain many highly cohesive nodes and support the leadership roles played by nurses and their communication activity in the ICU, as previously shown by the social network analysis that mapped operating rooms [16].

As the connectivity threshold rose to 150 and 180 min, focusing on highly communicative individuals in the ICU, a few non-nursing professions emerged at the periphery of the communication structure, whereas the core group remained a single cluster consisting of nurses (Figure 2E and Figure 2F). This suggests that high-volume communication in the ICU occurs predominantly as intraprofessional interactions among nurses.

**Figure 3:**
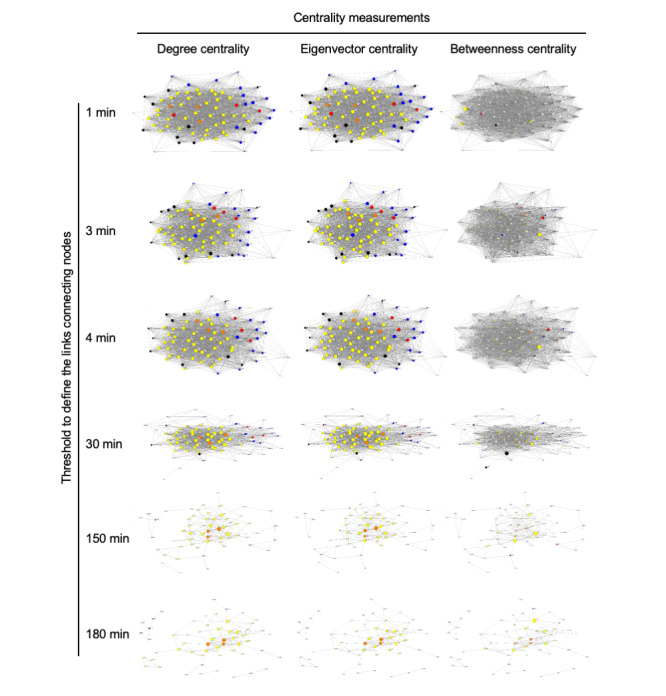
Centrality measurements of the social network in the active face-to-face interactions between ICU healthcare professionals. Three different centrality measures (degree of centrality, eigenvector centrality, and betweenness centrality) were used. Nodes represent individual ICU staff: blue and red, doctors; yellow and orange, nurses; black, other professions. The top 3 doctors and nurses who actively interacted the most are denoted by red and orange nodes, respectively as shown in Figure 2. Threshold values used to define those links connecting the nodes are 1, 3, 4, 30, 150, and 180 minutes.

To further substantiate our finding about the roles played by ICU nurses, we performed centrality measurements of the social network (Figure 3). We employed multiple centrality measurements such as the degree of centrality, eigenvector centrality, and betweenness centrality, in an attempt to identify the most prominent people in the network from different angles. By gradually changing the threshold from 1 to 180 min, different layers of the network in the ICU emerged, thereby demonstrating that nurses were centrally positioned at the core in all 3 centrality measurements (Figure 3), which confirmed the prominent roles of nurses in the communication network in the ICU.

## Discussion

By utilizing wearable sociometric sensor badges, the present study visualized the spatiotemporal distributions of the face-to-face interactions between health care professionals in the ICU. The social network analysis performed on the datasets collected by the wearable sensors also visualized the structures of the interprofessional interactions in the ICU, revealing the pivotal role played by nurses in the interaction network. The present study has extended our previous feasibility study of wearable sensors, which demonstrated not only that the interaction intensities changed periodically at 8-hour intervals, thereby reflecting the 3-shift changeover schedule of ICU workers (day, evening, and night shifts) [6], but also that the intensities of the interactions positively correlated with patient severity as measured by the APACHE II scores [6]. Similar sociometric wearable sensor technologies have been used to study the interactions between health care professionals, patients, and caregivers in a general pediatric hospital ward in Italy [17]; interactions between nurses at a surgical ICU at a university hospital in the US [18]; and interactions between doctors and nurses in the emergency department at university hospital in Italy [19]. In this way, the feasibility of the wearable sensors in monitoring human interactions in health care settings has been supported by multiple independent studies including ours [6].

Social network analysis has been used to study the relationships between individuals and the structures of teams in hospitals and other organizations [20]. Previous social network analysis studies performed in the field of multidisciplinary medical care have shown the important roles played by nurses. One study on operating room staffing identified a core/periphery network structure, in which nurses not only constituted the majority of the core members, but also led the multidisciplinary operating teams [16]. Another study on small ICU medical teams examined the formation of multidisciplinary communication networks, in which a doctor was at the center of one team network, while a nurse was at the center of another [21]. Yet another study on ICUs demonstrated that nurses represent an important group of influential individuals who create the central workplace culture [22]. The majority of previous social network analysis studies [16,20,21] used manual methods, such as surveys and observations, to collect connectivity data. The present study, which uses wearable sensors to continuously collect objective data, has substantiated the findings of previous social network analysis reports [16,20,21], namely that nurses play the leading role in such interprofessional communication networks. This conjuncture has been further corroborated by a series of centrality measurements of the network, including degree of centrality to describe the number of connections to a node; betweenness centrality to evaluate a node’s ability to connect others to one another; and eigenvector centrality to assess a node’s importance while simultaneously considering the importance of its neighboring nodes [13,14].

The nurse-to-physician ratio in ICUs might affect the patterns of social communication networks as more nurses could lead to their domination of communications. In the present study, the nurse-to-physician ratio in our ICU was 2.6, which appears to be higher than the estimated national average value of approximately 2 that we calculated from the publicly available data regarding ICUs in university hospitals in Japan. A previous study that examined 8 ICUs in university hospitals in France showed the nurse-to-physician ratio ranging from 1.8 to 5.6 [23]. How the patient-to-nurse and patient-to-physician ratios affect patient outcomes in ICUs has been well studied [23,24]. It is of continuing interest to study how the different nurse-to-physician ratios in ICUs facilitate such interprofessional collaborations, which can impact patient outcome.

Interprofessional communication and interactions are believed to play the pivotal role in facilitating the multidisciplinary collaborative work carried out in hospitals [25,26]. Effective collaborations between health care professionals may improve health care outcomes [25,26]. However, clinical evidence supporting the hypothesis that clinical and social interventions designed to promote interprofessional communication and collaboration do impact health care outcomes remains scarce [3]. One systematic review that examined 9 randomized clinical trials only found weak evidence supporting the claim that health care outcomes are improved by interventions to facilitate interprofessional collaborations [3]. It has been suggested that implementing robust analytic methods to examine human interactions is necessary if strong evidence in clinical trials is to be obtained [3]. Social network analysis might offer one such analytic method for studying the interprofessional interactions between health care professionals.

Social network analysis helps us better understand and objectively evaluate the structures of communication networks in ICUs, identifying targets of intervention that facilitate interprofessional communication. Some of our social network analysis results show that a few individuals play key roles in connecting the core members (nurses) to peripheral non-nursing professions, thereby acting as communication network hubs from which the flow of information is centered. By promoting such leading roles, highly effective interventions for facilitating wider interprofessional communication and collaboration can be developed and implemented. Alternatively, social network analysis could be used to objectively evaluate how interventions aimed at promoting interprofessional communication might actually improve these social communication networks.

## Appendix

Multimedia Appendix 1 The spatial distribution of active face-to-face interactions in the ICU. The daily sum of the lengths of the active interactions (persons X mins) was mapped to the floor layout of the ICU from days 1 to 30. Both inter- and intraprofessional interactions are included.

Multimedia Appendix 2 The spatial distribution of active face-to-face interactions involving nurses in the ICU. The daily sum of the lengths of the active interactions (persons X mins) was mapped to the floor layout of the ICU from days 1 to 30. The nurse-to-nurse and nurse-to-other profession interactions are both included.

Multimedia Appendix 3 The spatial distribution of active face-to-face interactions involving doctors in the ICU. The daily sum of the lengths of the active interactions (persons X mins) was mapped to the floor layout of the ICU from days 1 to 30. The doctor-to-doctor and doctor-to-other profession interactions are both included.

## Abbreviations

ICU: intensive care unit
